# A Rare Case of Cardiac Metastatic Uterine Intravenous Leiomyomatosis

**DOI:** 10.3389/fcvm.2022.871983

**Published:** 2022-04-26

**Authors:** Xun Wu, Feng Li, Chukwuemeka Daniel Iroegbu, Chengming Fan, GuoBao Song

**Affiliations:** Department of Cardiovascular Surgery, The Second Xiangya Hospital, Central South University, Changsha, China

**Keywords:** tricuspid chordae, intravenous leiomyomatosis, uterine leiomyomas, operation treatment, combined surgery

## Abstract

Intravenous leiomyomatosis (IVL) is a distinct uterine leiomyoma, even rare when combined with intracardiac invasion. Although leiomyomas are histologically benign, intracardiac metastasis may cause circulatory failure and death. Herein, we report a 55-year-old woman with a tricuspid chordae mass on echocardiography. Subsequently, gynecological ultrasonography revealed that the patient had masses in the ovaries, internal iliac vein, and inferior vena cava. The patient successfully underwent resection of the tricuspid chordae tendinea mass and implantation of the tricuspid annuloplasty ring. The patient underwent inferior vena cava, common iliac vein, hysterectomy, and bilateral adnexectomy after 4 months. To our knowledge, the present study is the first reported case with such a rare combination.

## Introduction

Intravenous leiomyomatosis (IVL) is a distinct uterine fibroids type, even rare when combined with intracardiac metastasis. Its primary feature, uterine benign leiomyoma formation, remains a characteristic hallmark for the disease. In addition, other typical IVL features include pelvic and intravenous masses. Notably, only 0.1% of cases become IVL disease (1). Although histologically benign, it possesses malignant tendencies. The tumor usually grows along blood vessels and extends toward the iliac vein and the inferior vena cava. It extends toward the right heart cavity and the main pulmonary artery in extreme cases. When the right heart is involved, the lesions have a “crutch-head” or “snake-head” appearance (2). Presently, fewer than 300 IVL cases have been reported, with relatively 100 intracardiac invasion cases (3). If not treated, intracardiac IVL can cause circulatory failure or death (4).

The study herein is a 55-year old female diagnosed with tricuspid chordal leiomyoma due to tricuspid valve regurgitation. The patient successfully underwent tricuspid chordectomy and valvuloplasty. Four months later, the inferior vena cava, common iliac vein, uterus, and bilateral appendages were removed. To our knowledge, this study is the first reported case with such a rare combination involving the tricuspid valve chordae tendineae.

## Case Report

A 55-year-old female patient was admitted to the hospital given a tricuspid mass lesion was found following a routine physical examination. The patient’s medical history recorded no heart palpitations, breathing difficulties, or other symptoms. A tricuspid murmur was found on cardiac auscultation. Transthoracic echocardiography showed a small tumor beneath the tricuspid valve with moderate tricuspid regurgitation. Biochemical indicators, such as blood and tumor markers, were within the normal range.

**FIGURE 1:**
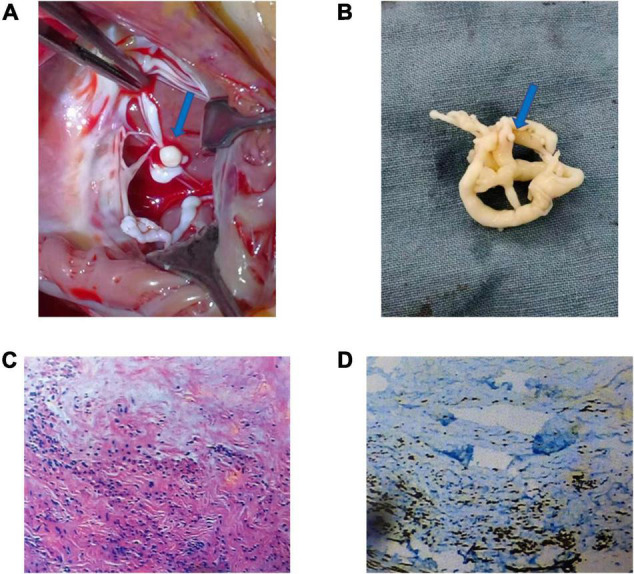
Morphology, hematoxylin and eosin (H&E) staining, and immunohistochemical staining of the mass of tricuspid chordae tendineae: Generally, the tumor is a 20 × 15 × 10 mm mushroom-like tumor **(A,B)** (blue arrow). The tumor comprises spindle cells, rare nuclear splits, necrosis of tumor – central tumor cells, and immunohistochemical HHF35 positive **(C,D)**.

A median sternotomy with extracorporeal circulation (CPB) and mild hypothermia was used to perform intracardiac tumor resection. During the operation, an isolated 20 × 15 × 10 mm mushroom-like tumor was attached to the anterior leaflet chordae of the tricuspid valve (Figure 1A). The tumor and chordae tendineae were excised (Figure 1B), followed by the implantation of artificial chordae tendineae and a tricuspid annuloplasty ring. Intraoperative transesophageal echocardiography confirmed that the tumor was removed entirely with restored tricuspid valve function. The histopathology report showed that the Ki-67 positive rate in the immunohistochemical analysis was 1%. The smooth muscle actin (SMA), Desmin, Vimehtin, and HHF35 were positive, while the Leucoyte common antigen (LCA), S100, CK, and PAS were negative (Figures 1C,D).

**FIGURE 2:**
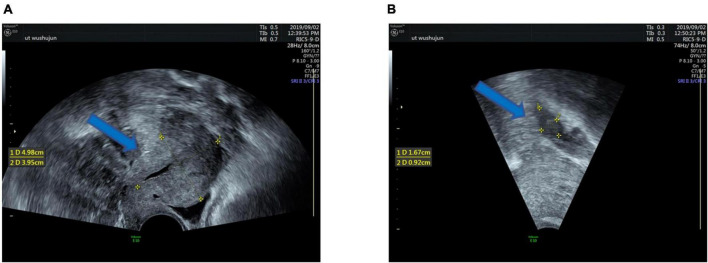
Clinical imaging performance: Ultrasound shows multiple uterine fibroids **(A)**, the maximum 4.98 × 1.95cm size (blue arrow). Ultrasound showing a 1.67 × 0.92 cm right ovarian mass **(B)** (blue arrow).

**FIGURE 3:**
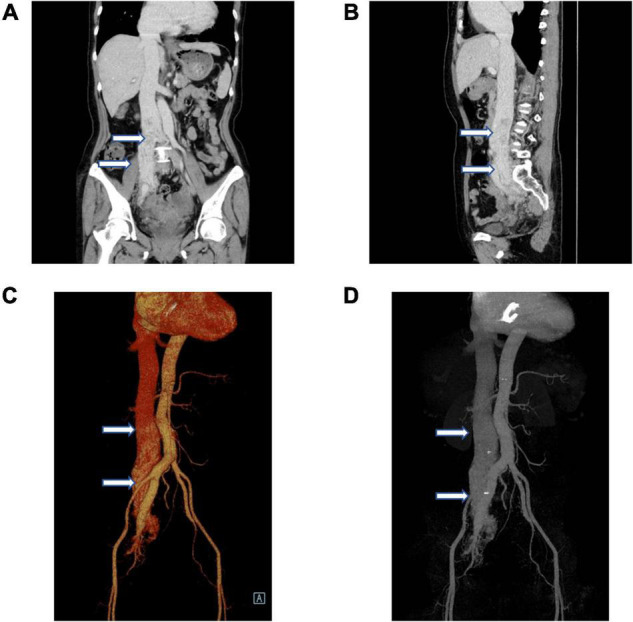
Vascular computer angiography (CTA) and 3D reconstruction show **(A–D)** the mass in the posterior wall of the uterine fundus extends to the pelvic veins, bilateral internal iliac veins, and inferior vena cava. The tumor did not invade the superior segment of the inferior vena cava and the heart (white arrows).

Given the intravenous leiomyomatosis possibilities, gynecological ultrasound was performed and found multiple uterine fibroids and ovarian tumors. Also, lumenal fluid and masses on the uterine fundus posterior wall, extending toward the bilateral internal iliac veins and the inferior vena cava, were found (Figures 2A,B). In addition, computer angiography (CTA) showed that the intravenous leiomyoma involved the right common iliac vein and the inferior vena cava (lower portion), extending toward the renal vein. There was no prominent involvement in the heart and the upper inferior vena cava segments (Figures 3A–D).

**FIGURE 4:**
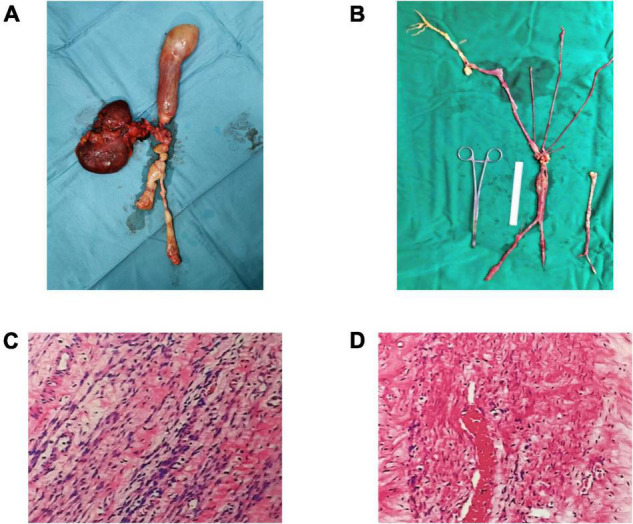
Leiomyoma specimens taken from the uterus, iliac vein, and inferior vena cava **(A,B)**. Under the microscope, the cells in the specimen are spindle-shaped, with no atypia and less mitosis **(C,D)**.

The patient underwent surgical procedures on the right common iliac vein, inferior vena cava, uterus, and bilateral salpingo-oophorectomy four-month postoperatively (Figures 4A,B). The histopathological report showed that the tissue was venous leiomyoma with abundant tumor cells, no obvious heterogeneity of tumor cells, and rare mitoses (Figures 4C,D). Ki-67 positive rate immunohistochemical analysis was 20%. The SMA, Desmin, H-caldesmon, Vimentin, and CD34 were positive, while the LCA, S100, CD117, and HMB45 were negative.

## Discussion

Echocardiography is the preferred imaging method when the patient has clinical symptoms related to the heart. Echocardiography is non-invasive and has no known risks or side effects. Echocardiography can effectively describe the relationship between the tumor and the heart cavity, valve, and other cardiac apparatus. Echocardiography can also highlight the neoplastic attachment site(s). It is essential to assess the tumor’s impact on valve function (5).

The patient’s heart color on the Doppler ultrasound showed a heterogeneous tricuspid valve echo. Thus, vegetation, thrombus, or cardiac tumor were suspected to be the diagnostic differentials. Given that the patient has no recent history of infection, fever, or drug use, the neoplastic chordae of the tricuspid valve was merely considered. Under ultrasound images, a thrombus can be seen throughout the cardiac cycle. The thrombus is usually close to the damaged myocardium and the congested area. Thrombus formation usually occurs with acute surgery cases, indwelling central catheters, or chronic diseases, particularly at the superior vena cava junction, and the right atrium (6). The patient has no history of myocardial damage or chronic disease. Therefore, the possibility of thrombus attaching to the tricuspid chordae was not considered.

Myxoma is the most prevalent cardiac tumor. Myxomas are highly mobile, usually confined to the heart chambers, and often attached to the atrial septum (7). Thus, determining its origin and attachment site can help distinguish intracardiac leiomyomas and myxomas. There have been no reports of myxoma originating from the chordae of the tricuspid valve. Hence, metastatic tumors, such as renal cell carcinoma, liver cancer, and adrenal tumors, should be considered. Notably, the patient has no other primary tumors.

Interestingly, IVL is a rare, benign, and smooth muscle tumor only seen in women. The pelvic and venous masses in a typical IVL are continuous. Though its pathogenesis is unclear, possible theories include its origin from blood vessel wall (smooth muscle) or uterine fibroids invasion into the uterine vein with significant widespread (8). The IVL may grow along blood vessels, extending to the iliac vein, inferior vena cava, and even the heart (8). There have been reports that IVL spontaneously migrates toward the heart through the bloodstream after hysterectomy. However, in this case, the intracardiac metastasis from the uterine IVL was isolated and attached to the anterior tricuspid valve chordae. There is no evidence that the tumor occurred in the inferior vena cava, internal iliac vein, or ovarian vein. It is worth noting that the intracardiac tumor, in this case, is in the tricuspid valve chordae. Therefore, it could have developed in the heart.

Surgical treatment of IVL with intracardiac manifestations: In the case herein, the tumor was resected *via* the right atrium after a circulatory interruption to enable adequate tricuspid tendon exposure and replacement. Given that cardiovascular-gynecological combination therapy is highly invasive, with the bleeding risk associated with systemic heparinization, we opted for a two-stage surgical treatment strategy.

To our knowledge, this study is the first reported case with such a rare combination seen in an elderly female with no evidence of benign metastatic leiomyoma. A related report described an asymptomatic 24-year-old female who denied solitary genital leiomyoma or reproductive system surgery, presenting as an intracardiac space-occupying lesion (9). This case has broadened the scope and IVL understanding with combined intracardiac involvement.

## Data Availability Statement

The original contributions presented in the study are included in the article/supplementary material, further inquiries can be directed to the corresponding author.

## Ethics Statement

Written informed consent was obtained from the individual(s) for the publication of any potentially identifiable images or data included in this article.

## Author Contributions

XW and FL contributed equally to the work. GS, XW, and FL contributed to the conception and design of the study. GS, XW, FL, CI, and CF wrote sections of the manuscript. All authors contributed to manuscript revision, read, and approved the submitted version.

## Conflict of Interest

The authors declare that the research was conducted in the absence of any commercial or financial relationships that could be construed as a potential conflict of interest.

## Publisher’s Note

All claims expressed in this article are solely those of the authors and do not necessarily represent those of their affiliated organizations, or those of the publisher, the editors and the reviewers. Any product that may be evaluated in this article, or claim that may be made by its manufacturer, is not guaranteed or endorsed by the publisher.
